# Leveraging National Claims and Hospital Big Data: Cohort Study on a Statin-Drug Interaction Use Case

**DOI:** 10.2196/29286

**Published:** 2021-12-13

**Authors:** Aurélie Bannay, Mathilde Bories, Pascal Le Corre, Christine Riou, Pierre Lemordant, Pascal Van Hille, Emmanuel Chazard, Xavier Dode, Marc Cuggia, Guillaume Bouzillé

**Affiliations:** 1 Université de Lorraine Centre Hospitalier Régional Universitaire de Nancy Centre national de la recherche scientifique, Inria, Laboratoire lorrain de recherche en informatique et ses applications Nancy France; 2 Inserm, Laboratoire Traitement du Signal et de l'Image - UMR 1099 Centre Hospitalier Universitaire de Rennes Université de Rennes 1 Rennes France; 3 Pôle Pharmacie Service Hospitalo-Universitaire de Pharmacie Centre Hospitalier Universitaire de Rennes Rennes France; 4 Laboratoire de Biopharmacie et Pharmacie Clinique Faculté de Pharmacie Université de Rennes 1 Rennes France; 5 Centre Hospitalier Universitaire de Rennes, Inserm, Ecole des hautes études en santé publique Institut de recherche en santé, environnement et travail, UMR_S 1085 Université de Rennes 1 Rennes France; 6 Centre d'Etudes et de Recherche en Informatique Médicale EA2694 Centre Hospitalier Universitaire de Lille Université de Lille Lille France; 7 Centre National Hospitalier d'Information sur le Médicament Paris France; 8 Department of Pharmacy Hospices Civils de Lyon University Hospital Lyon France

**Keywords:** drug interactions, statins, administrative claims, health care, big data, data linking, data warehousing

## Abstract

**Background:**

Linking different sources of medical data is a promising approach to analyze care trajectories. The aim of the INSHARE (Integrating and Sharing Health Big Data for Research) project was to provide the blueprint for a technological platform that facilitates integration, sharing, and reuse of data from 2 sources: the clinical data warehouse (CDW) of the Rennes academic hospital, called eHOP (entrepôt Hôpital), and a data set extracted from the French national claim data warehouse (Système National des Données de Santé [SNDS]).

**Objective:**

This study aims to demonstrate how the INSHARE platform can support big data analytic tasks in the health field using a pharmacovigilance use case based on statin consumption and statin-drug interactions.

**Methods:**

A Spark distributed cluster-computing framework was used for the record linkage procedure and all analyses. A semideterministic record linkage method based on the common variables between the chosen data sources was developed to identify all patients discharged after at least one hospital stay at the Rennes academic hospital between 2015 and 2017. The use-case study focused on a cohort of patients treated with statins prescribed by their general practitioner or during their hospital stay.

**Results:**

The whole process (record linkage procedure and use-case analyses) required 88 minutes. Of the 161,532 and 164,316 patients from the SNDS and eHOP CDW data sets, respectively, 159,495 patients were successfully linked (98.74% and 97.07% of patients from SNDS and eHOP CDW, respectively). Of the 16,806 patients with at least one statin delivery, 8293 patients started the consumption before and continued during the hospital stay, 6382 patients stopped statin consumption at hospital admission, and 2131 patients initiated statins in hospital. Statin-drug interactions occurred more frequently during hospitalization than in the community (3800/10,424, 36.45% and 3253/14,675, 22.17%, respectively; *P*<.001). Only 121 patients had the most severe level of statin-drug interaction. Hospital stay burden (length of stay and in-hospital mortality) was more severe in patients with statin-drug interactions during hospitalization.

**Conclusions:**

This study demonstrates the added value of combining and reusing clinical and claim data to provide large-scale measures of drug-drug interaction prevalence and care pathways outside hospitals. It builds a path to move the current health care system toward a Learning Health System using knowledge generated from research on real-world health data.

## Introduction

The secondary use of health care data offers the opportunity to conduct observational studies in real life [1-3]. Indeed, hospital clinical data warehouses (CDWs) supply fine-grained information from electronic health records (EHRs), such as laboratory test results and drug administration, but are restricted to hospitalized patients. Conversely, National claim databases offer limited information (eg, drug reimbursement and health care consumption data), but on a large part of the population. Therefore, matching the data from these 2 different databases could be informative, but it is also challenging. Patients existing in the 2 databases should be correctly identified using appropriate record linkage methods. The first option is deterministic record linkage that relies on the presence of a unique common identifier or a combination of different variables used as a key to join tables [4]. More complex rules to link records can also be added, such as an acceptable distance between string variables or between dates. The second option is probabilistic record linkage that is based on a model to assess the discriminative power of each variable used in the record linkage strategy. The result is the probability that an entity in the first database is the same entity in the second database [5,6]. Several studies have demonstrated that in most cases, probabilistic approaches give better results than deterministic methods [7-10]. However, the choice of record linkage also heavily depends on the characteristics of the 2 databases to be linked. The quality of the data used in the record linkage is an especially important factor. Indeed, if high quality data (eg, few missing values) are available, deterministic methods can achieve good results and are easier to develop [11].

In France, the national health database, Système National des Données de Santé (SNDS), [12] links the nationwide outpatient claim database (Système national d’information inter-régimes de l’Assurance maladie), the national discharge database (Programme de Médicalisation des Systèmes d’Information [PMSI]), and the Epidemiology Centre of Medical Causes of Death (CepiDC; vital status data) database. Rennes academic hospital (Centre Hospitalier Universitaire de Rennes) uses eHOP (entrepôt Hôpital) [13], a CDW that includes EHR and discharge data on all stays in this hospital. Linking SNDS and eHOP is a promising strategy to analyze patient care trajectories. However, legal, methodological, and technical barriers still remain. Health data are sensitive, and in France, their use is regulated by the European General Data Protection Regulation [14]. Therefore, studies based on the use of health data entail various regulatory steps, such as the scientific evaluation of the project and the patient information material and the assessment of the impact on data protection. In France, the use of SNDS data for external research requires the development of a data repository that complies with the strict security specifications to host the SNDS sample for the study.

In this context, the aim of the INSHARE (Integrating and Sharing Health Big Data for Research) project was to provide the blueprint for a technological platform (INSHARE platform) that facilitates data integration, sharing and reuse by following the FAIR (findability, accessibility, interoperability, and reusability) Guiding Principles [15]. This work demonstrates through a use case in pharmacovigilance how the INSHARE platform can support health big data analysis.

Our use case focused on statin consumption and statin-related drug–drug interactions (DDIs). Indeed, 36.9% [16] of French people aged 34 to 65 years have hypercholesterolemia, and statins are the most prescribed lipid-lowering treatment drugs in France [17]. The current European treatment guidelines [18] recommend statins as the first-choice drug for hypercholesterolemia management. However, 10% to 25% of patients treated with statins experience muscle side effects [19], including rhabdomyolysis (incidence: 1-3 in 100,000 persons per year) [20]. Statin-induced rhabdomyolysis is related to DDIs in 60% of cases [20], which suggests that avoiding DDIs has an important role in reducing statin adverse events. Because of their wide use and DDI potential, statins are an interesting study topic to assess the value of our technological platform for clinical data reuse. Moreover, literature data indicate that DDIs are preventable, but this is hindered by the clinicians’ lack of easy access to comprehensive information. Indeed, health care delivery is fragmented across the system and this creates an environment susceptible to medication-related issues [21]. Polypharmacy has been associated with higher risk of DDIs and adverse drug events [22], and subsequently, with drug-related deaths in hospitals [23]. Therefore, it is important to precisely characterize the individual care pathways within the health care system using aggregated medical data.

Here, we present the technical aspects of the INSHARE platform and the methods and results of the care pathway analysis in patients with statin-drug interactions.

## Methods

### Data Sources

#### Drug Database: Thériaque

Thériaque is a comprehensive dynamic knowledge database that provides exhaustive information on approved and marketed drugs [24]. It contains highly structured information on each drug, such as indications, contraindications, and DDIs and their severity level. Each drug is referenced according to 3 mapped classifications: Unité Commune de Dispensation, the medication-dispensing unit used by the French hospital information system; Code Identifiant de la Présentation, the drug package identifier used by French community pharmacies; and Anatomical Therapeutic Codification, which is based on the active component or components of each drug.

#### French Claim Database: SNDS

In France, the SNDS is a national claim data warehouse that covers 98.8% of the entire French population [25]. It contains data from outpatient care, such as medical consultations and drug deliveries by community pharmacies, and data from inpatient care, such as diagnosis and procedures performed during a stay in a private- or public-sector hospital. Each reimbursement of outpatient care is recorded at the individual level in a specific data mart called Datamart de Consommation InterRégime [12]. Data on inpatient care also are recorded at the individual level in an annual national discharge database called PMSI that is similar to the diagnosis-related groups. Individual data are deidentified and pseudonymized allowing the linkage, thanks to a unique identifier, between inpatient data (PMSI database) and outpatient data (Datamart de Consommation Inter Régime). This claim data warehouse has been previously described [12].

We used a data set extracted from the SNDS database that included all patients discharged after at least one hospital stay at Rennes academic hospital between 2015 and 2017. Owing to the redundancy of information contained in the PMSI database, hospital stays following the primary diagnosis were excluded (eg, stays for chemotherapy, radiotherapy, dialysis, apheresis, blood transfusion and hyperbaric oxygen therapy). All inpatient and outpatient data in the 12 months before each hospital stay were extracted.

Data were extracted from the national SNDS database by a French national health insurance manager outside of this study workflow.

#### CDW: eHOP

eHOP is the CDW developed and deployed at Rennes academic hospital [13]. It collects administrative and clinical data from EHRs, both unstructured (eg, clinical notes) and structured (eg, drugs, laboratory results). Data are deidentified and a unique anonymous identifier allows the linkage among hospital stays of a given patient. The eHOP CDW currently allows for searching from 80 million unstructured data and 430 million structured elements. All these data are collected from EHRs and cover more than 1.4 million patients.

The data set from the eHOP database included patients according to the same criteria used for the SNDS data: all data on hospital stays at Rennes academic hospital between 2015 and 2017. For this study, we used the following structured data:

1. Demographic data
2. Drug administered (Common Unit of Dispensation, UCD and date of administration)
3. PMSI data: *International Classification of Diseases, Tenth Revision (ICD-10)* codes, procedure codes, mortality, length of stay, etc.
4. Laboratory results described with a local terminology.

### Record Linkage Procedure

**Figure 1:**
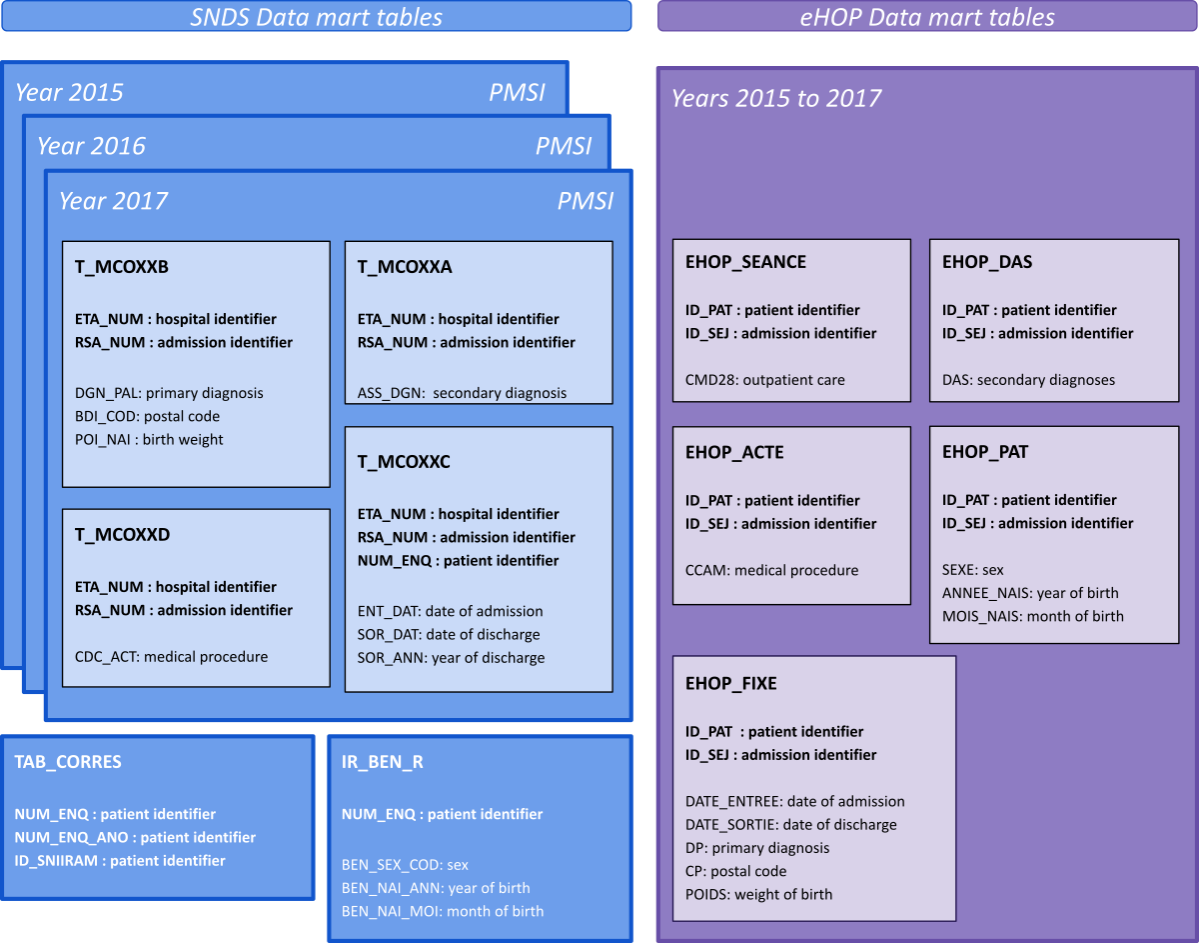
SNDS data mart tables (in blue), including PMSI tables, and eHOP data mart tables (in purple) with the different variables from the 2 data sources used for the linkage procedure. eHOP: entrepôt Hôpital; PMSI: Programme de Médicalisation des Systèmes d’Information; SNDS: Système National des Données de Santé.

**Figure 2:**
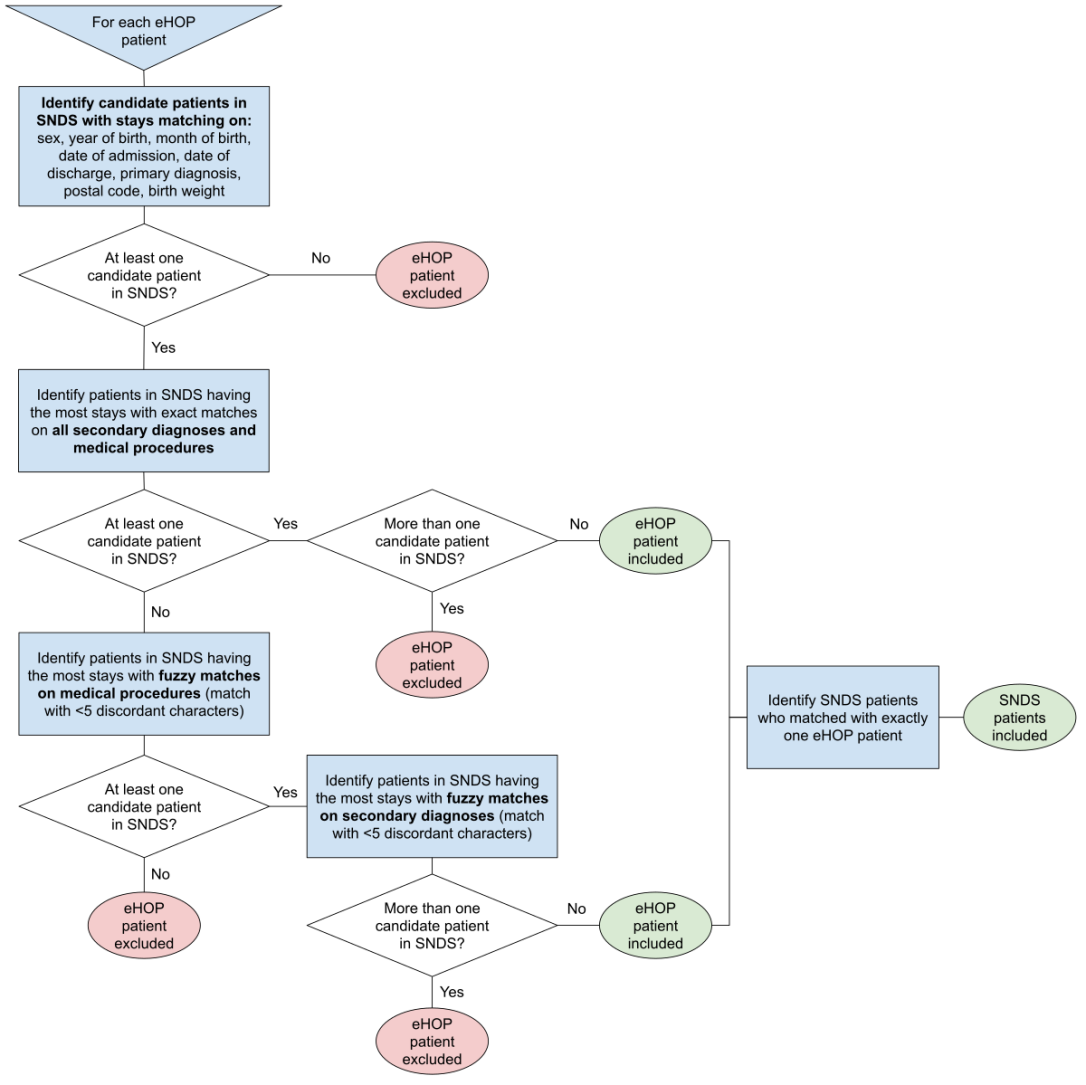
Decision tree for the record linkage procedure. eHOP: entrepôt Hôpital; SNDS: Système National des Données de Santé.

As no unique patient identifier is available to link SNDS and eHOP data because of regulatory issues, we developed a semideterministic record linkage method based on PMSI variables that are common between the SNDS data source and the eHOP CDW data source (Figure 1). PMSI data are available from all French hospitals and are produced in a standardized way by each hospital. Once deidentified, PMSI data feed the nationwide PMSI database. This database is then integrated in the SNDS database to link PMSI data with claim data. In theory, PMSI data from the SNDS and hospitals should be exactly the same. However, during the preliminary work, we identified some discrepancies concerning *ICD-10* and procedure codes between these data sources. Therefore, we incorporated some fuzzy logic in the record linkage algorithm to solve inconsistencies. The algorithm is illustrated in Figure 2. Specifically, *ICD-10* codes comprise between 3 and 6 characters, but we kept only the first 4 characters. Procedure codes comprise 7 characters, and we kept all 7. We merged *ICD-10* and procedure codes in alphabetical order in a unique string for each stay. We then tested different Levenshtein distance thresholds to consider a match between sets of codes (the distribution of the Levenshtein distances for the *ICD-10* codes and procedure codes is provided in Multimedia Appendix 1, Table S1). We identified a threshold of 5 as the best choice for both *ICD-10* and procedure codes. For the final matching, first we assessed whether a patient had at least one exact match. This was considered as the exact match if the other patients were fuzzy matches. If we did not find any exact match, we kept the fuzzy match first looking at procedure codes. If a patient had several exact matches or several fuzzy matches, we kept the one with the most fuzzy matches on *ICD-10* codes. The remaining patients with several matches were considered as duplicates and were excluded from the linkage results.

We also had to solve specific cases concerning twins who do not have an individual identifier (NUM_ENQ) in the PMSI. Indeed, the same identifier (NUM_ENQ) is shared by twins of the same sex [12]. Thus, it was impossible to link their SNDS records with their records in eHOP. We chose to exclude twin patients from the record linkage results. The complete algorithm is available in Multimedia Appendix 1, Figure S1.

We assessed the linkage effectiveness by calculating the rate of SNDS and eHOP patients who could be matched in the other data set. We also describe some characteristics of the following groups: patients who were matched between data sources, and patients from the SNDS and eHOP data sets who could not be matched.

### INSHARE Platform

The INSHARE platform comprises 2 parts: a data repository to gather all kinds of data sources, and a computing infrastructure to perform data preparation, record linkage and analyses. The platform is available through Apache Mesos, a resource manager, to allow concurrent access to the computing server.

The data repository was the Apache Hadoop Distributed File System (HDFS) repository, and data were stored in parquet format files, with an appropriate stratification key. SNDS data sets were made available to us in CSV files that were stored in a specific folder in the server. We extracted the data needed from the eHOP CDW and the Thériaque databases with Spark SQL. This extraction step avoided repeating long queries in the CDW and overloading the production CDW used for other purposes.

We used the Spark distributed computing framework, version 2.3.4, for the data preparation, the record linkage procedure, and all use-case analyses.

**Figure 3:**
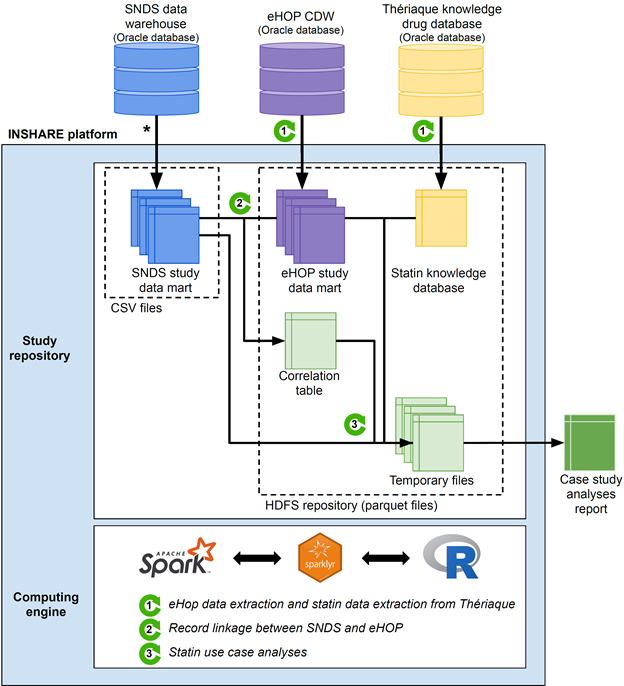
INSHARE platform and data processing workflow. CDW: clinical data warehouse; eHOP: entrepôt Hôpital; HDFS: Hadoop Distributed File System; INSHARE: Integrating and Sharing Health Big Data for Research; SNDS: Système National des Données de Santé.

We then accessed these data with Spark SQL that allowed us to merge data from the different sources in an efficient way and to perform all analyses. We used the R language as the script language, particularly the sparklyr package. The overall data processing is depicted in Figure 3.

We used a single node cluster: a CentOS 7 Unix server with 2 Intel Xeon 5122@3.6 GHz and 192 GB of RAM. Thus, we did not replicate the HDFS repository, and we executed the Spark master and slave nodes on the same machine.

### Use Case Study Design

We performed a cohort study on patients treated with statins prescribed by their general practitioners or during the hospital stay. We collected information on statins (Anatomical Therapeutic Codification classes C10AA, C10BA, and C10BX) and the statin-drug interactions from the Thériaque database. We classified statin intake as (1) community consumption if we found at least one statin delivery by a community pharmacy less than 1 month before hospitalization, and (2) hospital consumption if we found at least one statine administered during the hospital stay. Only the first hospital stay for each patient was retained for the use-case.

For each patient, we extracted the following features: sex, age at admission, the international nonproprietary name of the used statin, consumption of drugs potentially interacting with the used statin, DDI severity, admission via the emergency department, length of hospital stay, in-hospital death, laboratory results: creatine phosphokinase (CPK), creatinineaemia, glycemia, hemoglobin, kalemia, natremia, aspartate aminotransferase, alanine aminotransferase, hospital care burden (ie, diagnosis-related group severity).

We classified patients into 3 subgroups according to their statin consumption status: (1) patients treated with statins before and during their hospital stay, (2) patients treated with statins before admission, but not during the hospital stay, (3) patient who started taking statins in hospital without any statin treatment in the previous 12 months. We defined a statin-related DDI on the basis of the intake of a drug that reacts with the statin taken by that patient. All hospital drug administrations were considered during the index hospital stay, and all community deliveries were considered within 8 days before or after the statin delivery. According to the Thériaque database, we classified all statin-drug interactions into 3 levels of severity (level 1: contraindication, level 2: relative contraindication, level 3: precaution of use).

### Statistical Analyses

We described categorical variables as numbers and percentages, and quantitative variables as mean and SD for symmetrical distribution, and median with first and third quartiles (Q1–Q3), otherwise. We explored the association between patient characteristics or hospital stays and the occurrence of a statin-drug interaction with the Chi-square test (categorical variables) and one-way analysis of variance (quantitative variables). We built a logistic regression model to identify factors independently related to the occurrence of a statin interaction.

### Ethical Consideration

The record linkage and the use-case study were approved by the Commission nationale de l’informatique et des libertés (French Data Protection Agency or CNIL; N 2,206,739). According to French regulations, patients were informed about the use of their data, and no signed consent was required.

## Results

### Technological Results

#### INSHARE Overall Computing Performance

**Figure 4:**
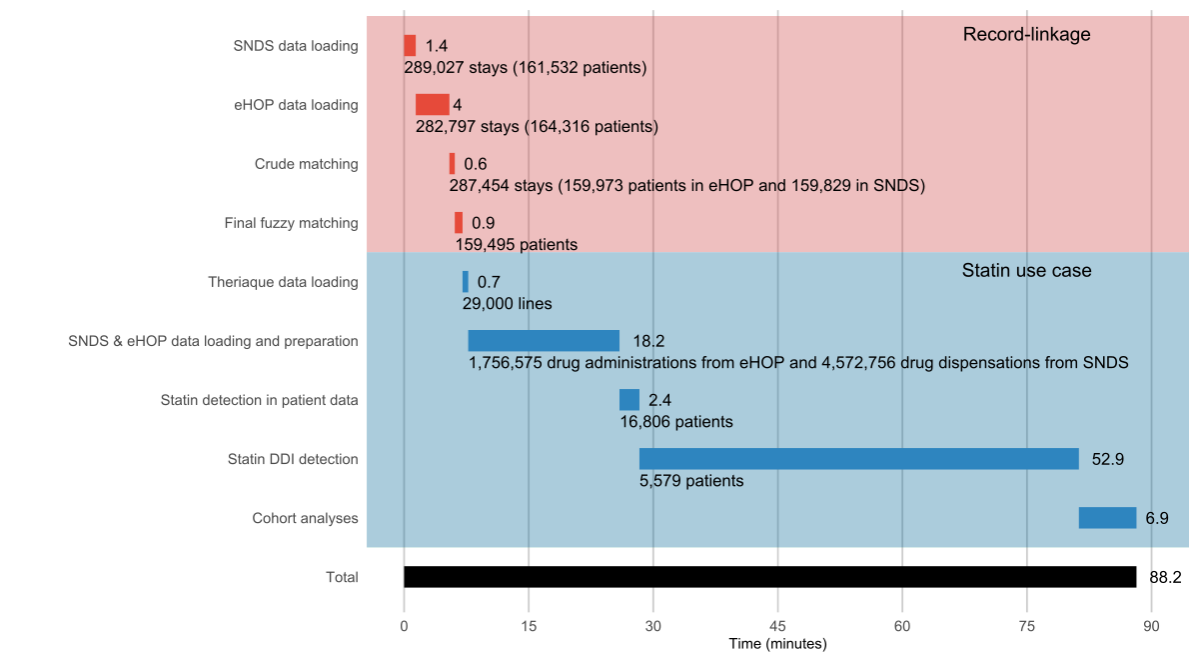
Time duration from data loading to the end of the use case-study analysis. DDI: drug–drug interaction; eHOP: entrepôt Hôpital; SNDS: Système National des Données de Santé.

The time needed for the record linkage procedure and statin use-case analysis was 88 minutes. The most time-consuming step was the detection of DDIs in the data of patients taking a statin. The time needed for each step is indicated in Figure 4.

#### Assessment of the Record Linkage Procedure

The SNDS and eHOP data sets included 161,532 subjects (278,341 stays) and 164,316 subjects (265,089 stays), respectively, who had at least one hospital stay at Rennes academic hospital between 2015 and 2017.

We successfully linked 159,495 patients (159,495/161,532, 98.74% and 159,495/164,316, 97.07% patients from the SNDS and eHOP data sets, respectively). We excluded from the linkage results 199 patients from the SNDS data set and 162 patients from the eHOP data set because their records were linked with more than one patient in the other data set. Patients who could not be linked were younger (median age of the unmatched patients from the eHOP and SNDS data sets: 22.3 and 27.6 years, respectively, compared with 48.4 years for matched patients). Moreover, women represented 51.35% (81,900/159,495) of all matched patients and 57.20% (2758/4821) and 18.52% (377/2037) of unmatched patients in the eHOP and SNDS data sets, respectively.

### Use Case Results

#### Statin-Taking Population

Of the 159,495 matched patients, we retained 16,806 patients with at least one statine delivery. Specifically, 8293 patients started statin treatment before admission and continued it during the hospital stay (community and hospital consumption), 6382 patients started statin treatment before admission but stopped at hospital admission (only community consumption), and 2131 patients started statins in the hospital (hospital initiation). The characteristics of the 3 subgroups are summarized in Table 1. Age (4651/6382, 72.88% and 6255/8293, 75.43% of patients aged ≥65 years) and unplanned hospitalization rate (2416/6382, 37.86% and 2434/8293, 29.35%) were similar in patients with only community consumption and patients with community and hospital consumption, respectively. Type of hospital care was similar in patients with community and hospital consumption and in patients with hospital initiation (4729/8293, 57.02% and 1072/2131, 50.31% of surgery, respectively). The percentage of patients aged ≥65 years and the rate of planned hospitalizations were lower in patients with hospital initiation than in the other 2 subgroups.

The most dispensed statin in all 3 subgroups was atorvastatin. Simvastatin, rosuvastatin and pravastatin each represented approximately 1 out of 5 prescriptions in patients with only community consumption. In the hospital, only 2 statins were available (atorvastatin and pravastatin).

**Table 1:** Patients’ characteristics according to their statin consumption.

					Only community consumption (n=6382), n (%)		Community and hospital consumption (n=8293), n (%)		Hospital initiation (n=2131), n (%)		*P* value
Sex (male)					3790 (59.39)		5431 (65.49)		1437 (67.43)		<.001
Age (≥65 years)					4651 (72.88)		6255 (75.43)		1192 (55.94)		<.001
Unscheduled admission					2416 (37.86)		2434 (29.35)		1155 (54.2)		<.001
**Type of care**											<.001
	Medical care				4576 (71.7)		3564 (42.98)		1059 (49.69)		
	Surgery				1806 (28.29)		4729 (57.02)		1072 (50.31)		
Chronic statin consumption (>3 months)					6075 (95.19)		7660 (92.37)		—^a^		<.001
**Statin type**											<.001
	Atorvastatin				2380 (37.29)		4632 (55.85)		1909 (89.58)		
	Fluvastatin				194 (3.04)		190 (2.29)		3 (0.14)		
	Pravastatin				1374 (21.53)		2004 (24.16)		183 (8.58)		
	Rosuvastatin				1145 (17.94)		1473 (17.76)		24 (1.13)		
	Simvastatin				1301 (20.39)		1540 (18.57)		24 (1.13)		
**Patients with statin-drug interactions**											
	**During community consumption**				1438 (22.53)		1815 (21.89)		—		<.001
		**DDI^b^ severity**									.07
			1	30 (2.09)		20 (1.10)		—			
			2	20 (1.39)		29 (1.60)		—			
			3	1404 (97.64)		1784 (98.29)		—			
	**During hospital consumption**				—		3215 (38.77)		585 (27.45)		<.001
		**DDI severity**									<.001
			1	—		72 (2.24)		10 (1.71)			
			2	—		143 (4.45)		58 (9.91)			
			3	—		3154 (98.10)		552 (94.36)			

^a^Not available.

^b^DDI: drug–drug interaction.

#### Statin-Drug Interaction Detection

We identified 5579 patients with potential statin-related DDIs. Overall, statin-drug interactions occurred more frequently during hospitalization than in the community (3800/10,424, 36.45% and 3253/14,675, 22.17%, respectively). The most severe DDIs (level 1) concerned 0.78% (82/10,424) of hospitalized patients.

Table 2 presents the hospital outcomes in patients with and without statin-drug interactions. Patients with statin-drug interactions were divided into 3 subgroups according to the place of DDI occurrence: (1) during community consumption (regardless of their hospital consumption), (2) during hospital consumption (regardless of their community consumption), or (3) during both community and hospital consumption. Statin-drug interactions occurring in hospital were associated with longer hospital stay, more severe pathology, and higher in-hospital mortality. The logistic regression model identified characteristics that were significantly related to the occurrence of statin-drug interactions: men older than 64 years of age, admitted for medical care for severe pathology, and longer length of hospital stay (Table 3).

Tables 4 and 5 present the frequency of patients according to their DDI severity and to the place of DDI occurrence and the details of the 5 most frequent drugs that interacted with statins according to the place of DDI occurrence.

**Table 2:** Characteristics of patients and hospital stays according to the place of the statin-drug interaction occurrence.

		Interaction only during community consumption (n=1779)	Interaction only during hospital consumption (n=2326)	Interaction during community and also hospital consumption (n=1474)	No interaction (n=11,227)	*P* value	
Sex (men), n (%)		1132 (63.63)	1521 (65.39)	1008 (68.39)	6997 (62.32)	<.001	
Age (≥65 years), n (%)		1394 (78.36)	1750 (75.24)	1215 (82.43)	7739 (68.93)	<.001	
Unscheduled admission, n (%)		698 (39.24)	926 (39.81)	544 (36.91)	3837 (34.18)	<.001	
**Type of care, n (%)**							<.001
	Medical care	1233 (69.31)	1149 (49.39)	787 (53.39)	6030 (53.71)		
	Surgery	546 (30.69)	1177 (50.6)	687 (46.61)	5197 (46.29)		
Length of stay (days), mean (SD)		8.3 (10.4)	11.9 (15.8)	8.4 (10.1)	7.6 (8.2)	<.001	
Intensive care unit admission, n (%)		94 (5.28)	742 (31.9)	260 (17.64)	2156 (19.2)	<.001	
**Diagnosis-related group severity, n (%)**							<.001
	1 (least severe)	1314 (73.86)	692 (29.75)	565 (38.33)	7070 (62.97)		
	2	177 (9.95)	673 (28.93)	388 (26.32)	2015 (17.95)		
	3	197 (11.07)	729 (31.34)	396 (26.87)	1692 (15.07)		
	4 (most severe)	91 (5.12)	232 (9.97)	125 (8.48)	450 (4)		
In-hospital mortality, n (%)		12 (0.67)	24 (1.03)	31 (2.1)	87 (0.77)	<.001	

**Table 3:** Factors related to the occurrence of a statin interaction.

			Odds ratio (95% CI)
Sex (male)			1.14 (1.04-1.25)
Age (≥65 years)			1.48 (1.34-1.62)
Unscheduled admission			1.08 (0.97-1.19)
Medical care			1.56 (1.43-1.71)
Length of stay (days)			1.03 (1.03-1.04)
**Diagnosis-related group severity**			
	1 (least severe)	1	
	2	1.18 (1.06-1.31)	
	3	1.27 (1.13-1.43)	
	4 (most severe)	1.51 (1.22-1.86)	

**Table 4:** Top 5 drugs interacting with statins during community consumption, along with the overall total for each security level.

Drug or statin		Rosuvastatin, n (%)	Simvastatin, n (%)	Atorvastatin, n (%)	Pravastatin, n (%)	Fluvastatin, n (%)
**Severity level: 1 (most severe)**						
	Cyclosporin (n=29)	17 (68)	12 (63.2)	0 (0)	0 (0)	0 (0)
	Sodium fusidate (n=8)	1 (4)	3 (15.8)	2 (50)	2 (100)	0 (0)
	Fenofibrate (n=6)	6 (24)	0 (0)	0 (0)	0 (0)	0 (0)
	Telithromycin (n=3)	0 (0)	1 (5.3)	2 (50)	0 (0)	0 (0)
	Clarithromycin (n=3)	0 (0)	3 (15.8)	0 (0)	0 (0)	0 (0)
	Total	25 (100)	19 (100)	4 (100)	2 (100)	0 (0)
**Severity level: 2**						
	Fenofibrate (n=23)	6 (85.7)	3 (13)	6 (54.5)	3 (100)	5 (83.3)
	Carbamazepine (n=15)	0 (0)	15 (65.2)	0 (0)	0 (0)	0 (0)
	Cyclosporin (n=6)	0 (0)	4 (17.4)	2 (18.2)	0 (0)	0 (0)
	Rifampicin (n=2)	0 (0)	1 (4.3)	1 (9.1)	0 (0)	0 (0)
	Bezafibrate (n=2)	1 (14.3)	0 (0)	0 (0)	0 (0)	1 (16.7)
	Total	7 (100)	23 (100)	11 (100)	3 (100)	6 (100)
**Severity level: 3 (least severe)**						
	Fluindione (n=898)	156 (26.5)	164 (13.1)	365 (22.2)	194 (25.7)	19 (22.9)
	Warfarin sodium (n=674)	114 (19.4)	101 (8)	287 (17.5)	152 (20.1)	20 (24.1)
	Amlodipine besylate (n=322)	0 (0)	322 (25.6)	0 (0)	0 (0)	0 (0)
	Sodium bicarbonate or sodium alginate^a^ (n=285)	51 (8.7)	58 (4.6)	110 (6.7)	57 (7.5)	9 (10.8)
	Sodium polystyrene sulfonate (n=225)	38 (6.5)	0 (0)	102 (6.2)	52 (6.9)	0 (0)
	Total	588 (100)	1256 (100)	1643 (100)	755 (100)	83 (100)

^a^Sodium bicarbonate-containing antacid.

**Table 5:** Top 5 drugs interacting with statins during hospital consumption, along with the overall total for each security level.

Drug or statin			Rosuvastatin, n (%)		Simvastatin, n (%)		Atorvastatin, n (%)		Pravastatin, n (%)		Fluvastatin, n (%)
**Severity level: 1 (most severe)**											
	Sodium fusidate (n=21)	3 (18.7)		0 (0)		14 (41.2)		4 (80)		0 (0)	
	Itraconazole (n=19)	0 (0)		2 (6.9)		17 (50)		0 (0)		0 (0)	
	Cyclosporin (n=15)	6 (37.5)		9 (31)		0 (0)		0 (0)		0 (0)	
	Erythromycin (n=12)	0 (0)		12 (41.4)		0 (0)		0 (0)		0 (0)	
	Fenofibrate (n=7)	7 (43.8)		0 (0)		0 (0)		0 (0)		0 (0)	
	Total	16 (100)		29 (100)		34 (100)		5 (100)		0 (0)	
**Severity level: 2**											
	Rifampicin (n=118)	0 (0)		11 (40.7)		107 (56.9)		0 (0)		0 (0)	
	Fenofibrate (n=56)	7 (58.3)		3 (11.1)		36 (19.1)		8 (61.5)		2 (100)	
	Daptomycin (n=30)	5 (41.7)		3 (11.1)		19 (10.1)		3 (23.1)		0 (0)	
	Isoniazid (n=9)	0 (0)		0 (0)		9 (4.8)		0 (0)		0 (0)	
	Cyclosporin (n=8)	0 (0)		5 (18.5)		3 (1.6)		0 (0)		0 (0)	
	Total	12 (100)		27 (100)		188 (100)		13 (100)		2 (100)	
**Severity level: 3 (least severe)**											
	Sodium polystyrene sulfonate (n=1142)	100 (19.7)		98 (9.4)		660 (18.7)		243 (20.2)		13 (16.5)	
	Warfarin sodium (n=894)	83 (16.3)		65 (6.2)		529 (14.9)		206 (17.1)		11 (13.9)	
	Fluindione (n=894)	92 (18.1)		88 (8.5)		504 (14.3)		196 (16.3)		14 (17.7)	
	Diosmectite (n=431)	42 (8.3)		31 (2.9)		259 (7.3)		93 (7.7)		6 (7.6)	
	Sodium bicarbonate or sodium alginate^a^ (n=421)	39 (7.7)		39 (3.7)		242 (6.8)		93 (7.7)		8 (10.1)	
	Total	508 (100)		1040 (100)		3531 (100)		1204 (100)		79 (100)	

^a^Sodium bicarbonate-containing antacid.

#### Link Between Statin-Drug Interaction and Laboratory Results

**Figure 5:**
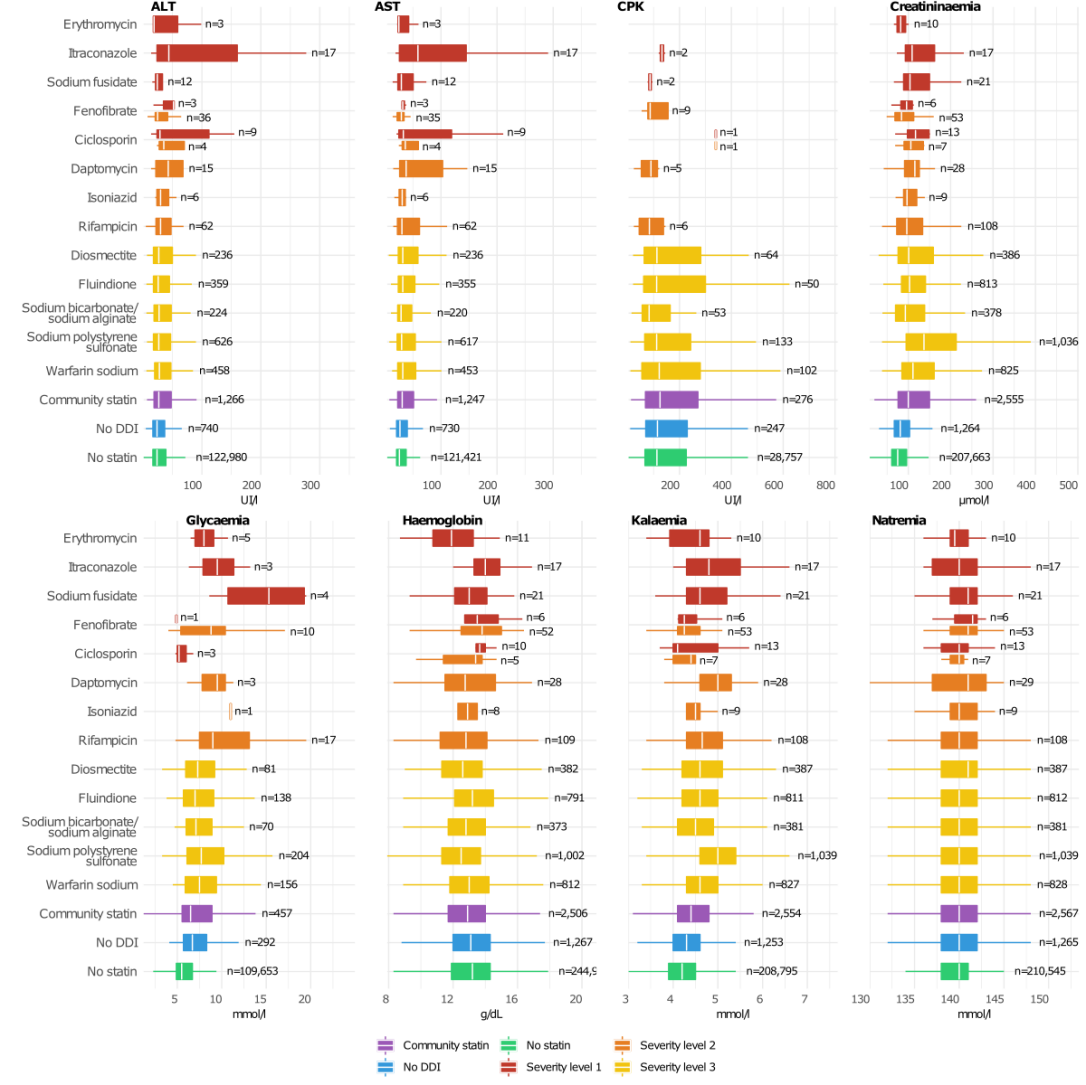
Boxplots of laboratory results for the top 5 DDIs of each statin. The 3 control groups are depicted in purple, blue and green. Boxplots in yellow, orange and red indicate the laboratory results of patients exposed to statin-related DDI with a level of severity of 3, 2 and 1 (the most severe). Patients can have more than one DDI, and they can be of different severity. Fenofibrate and cyclosporin have 2 boxplots because some of their DDIs are classified as level 2 and others as level 1. ALT: alanine aminotransferase; AST: aspartate aminotransferase; CPK: creatine phosphokinase; DDI: drug–drug interaction.

Figure 5 illustrates the link between the 5 most frequent drug interactions of each statin and the laboratory results. Overall, we observed little variations in laboratory values between patients with level 3 statin-drug interactions and patients without statins or taking statins but without DDI. However, glycemia was higher in patients in whom a potential statin interaction (level 1) with sodium fusidate, itraconazole, or erythromycin was detected. Similarly, kalemia and liver enzymes (alanine aminotransferase, aspartate aminotransferase) were altered in patients with a potential statin interaction (level 1) with itraconazole, or sodium fusidate, and with itraconazole, respectively. However, the sample sizes were too small (fewer than 20 patients for most laboratory data, particularly for CPK) to detect any significant variation.

## Discussion

### Strengths

#### Technical Work

To the best of our knowledge, this is the first study that successfully linked EHR data, through a CDW and claim data. However, there are some initiatives that integrate the 2 data types at the source into a common database [26,27].

The linkage process was efficient and generic enough to be applied to any data source that contains PMSI data. Our goal was to demonstrate that for data reuse purposes, it is possible to link fine-grained EHR data and claim data without a common patient identifier. Today, most hospitals have a CDW dedicated to research and fed with EHR data. Specifically, we used the eHOP CDW architecture that is currently the most widespread CDW type in France [13].

These 2 data sources can be bulky. For instance, the statin use-cases required to read and filter all drug administrations (n=13,125,574) and all drug dispensations (n=6,019,432) to identify patients to be included in the study were large. To ensure fast computation, we developed a computing framework based on Spark and HDFS that showed good performances even on our small single node cluster. These tools are widespread in the big data field, but they are still rarely used for data reuse in hospitals. According to Dolezel et al [28], their underuse, despite the massive amount of hospital data available, is explained by the lack of personnel with specific technological skills.

#### DDI Use Case

Our use-case study found a statin-drug interaction prevalence of 22.17% (3253/14,675) and 36.45% (3800/10,424), during community consumption and hospital consumption, respectively. Few studies have provided statin-drug interaction rates during primary care and hospital care for the same population. A Bulgarian study [29] reported statin-drug interaction prevalence rates of 26.1% at hospital admission (used as a proxy for primary care prescription) and 24.4% at discharge. Regarding primary care, this rate ranges from 6.9% [30] to 33% in a systematic review [31] on elderly patients. However, the definition of interaction varies among studies. This could be explained not only by the choice of drug database, as reported in the literature [32,33], but also by the focus on the most severe interactions. Our study took into account different severity levels, from precaution of use to contraindication, using the Thériaque database.

By comparing the places where interactions occurred (community or hospital), our study showed that the most severe interactions in the hospital led to more specialized and longer care, as previously reported [34]. This should be put in perspective with the larger number and types of drugs administered during hospital stays. Finally, we attempted to link DDIs and laboratory results and showed their potential impact on some laboratory parameters. Previous works reported the biological effects of some statin-drug interactions, such as (1) liver toxicity (elevated alanine aminotransferase or aspartate aminotransferase) by interaction with cyclosporin, (2) hyperkalemia [34] with itraconazole or erythromycin [35], and (3) hyperglycemia with fusidic acid [36]. These findings should be interpreted with caution because some of them could be because of the adverse effects of statins [37] or of the other drug, such as itraconazole.

### Limitations

#### Technical Work

The pairing procedure showed that the data life cycle introduced quality defects that explained the incomplete record linkage. We are still investigating the reasons for the match failures and how to explain quality data defects. The record linkage procedure could be improved using more sophisticated linkage strategies, such as probabilistic methods. However, our study concerned a specific case where data variables used for the record linkage procedure originated from the same source (ie, PMSI data produced by hospitals). Most of the unmatched patients were twins who could not be distinguished in the SNDS data, even by using more complex methods. We think that the deterministic approach is simpler to maintain and is more understandable for people who would like to use or adapt our algorithm for their own purpose.

#### DDI Use Case

DDI prevalence remains dependent on the chosen definition. In our study, these interactions were based only on the simple presence of a drug that could interact with statins and did not capture dose-dependency or patient-specific factors that might influence DDI definitions. Moreover, only information on dispensation was available for primary care (community consumption), whereas administrations were considered for hospital stay.

Despite the large cohort of patients over a 3-year period, our use case study found only 121 patients with a severity level 1 DDI, and among them only 5 had CPK data. This highlights the importance of the large sample size needed in pharmacoepidemiology and pharmacovigilance studies to detect rare adverse effects.

### Conclusions

This study demonstrates the added value of combining and reusing clinical and claim data to provide large-scale measures of DDI prevalence and care pathways outside hospitals. In a complex health care system that involves multiple care providers, transitions of care are often the source of medication discrepancies and DDIs [38]. Linking CDW and community data is a promising approach to identify gaps in the system.

Our approach also allows performing big data–driven analyses to generate new hypotheses. For instance, by linking laboratory data with DDIs, we demonstrated that our strategy allowed exploring potential biological variations associated with DDI exposure. However, because of the small patient samples with laboratory results and the exploratory design of the study, we did not want to infer any causal effect or clinical impact at this step. In this context, data reuse should be complementary to hypothesis-driven pharmacoepidemiological research, which is the appropriate way to confirm the plausibility of a given hypothesis generated using health data.

This builds the path to progress toward a Learning Health System, in which patient care is continuously improved using knowledge generated from research on real-world health data and clinical research [39].

Since the INSHARE project, we have extended this approach in the HUGOSHARE project in which we plan to analyze, using the Health Data Hub platform [40], the DDIs for a larger number of drug classes in a much bigger data set from SNDS and from the CDWs of 6 academic hospitals of the French western area. This may overcome the limitations of this study concerning the limited sample sizes for rare events with the aim to generate high quality hypotheses and to consider building predictive models.

Future medical technological developments may also consider enriching community pharmacy reimbursement data with other community data, such as community laboratory results or ambulatory visits. This might enable researchers to identify system vulnerabilities that result in medication errors slipping through the holes of the *Swiss Cheese Model of System Errors* [41,42].

## Appendix

Multimedia Appendix 1 Pseudocode describing the different steps of the record-linkage algorithm.

## Abbreviations

CDW: clinical data warehouse
CPK: creatine phosphokinase
DDI: drug–drug interaction
eHOP: entrepôt Hôpital
EHR: electronic health record
FAIR: findability, accessibility, interoperability, and reusability
HDFS: Hadoop Distributed File System
ICD-10: International Classification of Diseases, Tenth Revision
INSHARE: Integrating and Sharing Health Big Data for Research
PMSI: Programme de Médicalisation des Systèmes d’Information
SNDS: Système National des Données de Santé
