# Supplementary material for: Leveraging National Claims and Hospital Big Data: Cohort Study on a Statin-Drug Interaction Use Case
Source: JMIR Med Inform. 2021 Dec 13;9(12):e29286. doi: 10.2196/29286 (PMC8713098; doi:10.2196/29286)
Supplement: Multimedia Appendix 1 [file medinform_v9i12e29286_app1.docx]

## Appendix


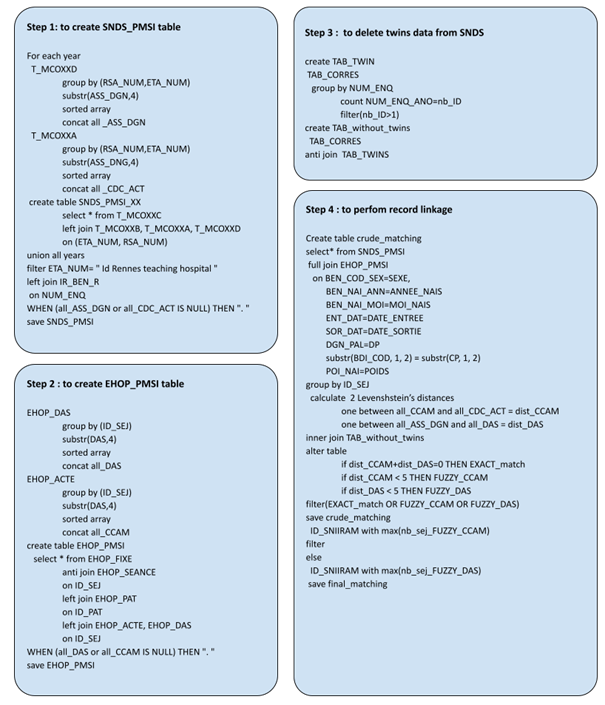


Figure S1. Pseudocode describing the different steps of the record-linkage algorithm.

Table S1. Distributions of Levenshtein distances for procedure and diagnostic codes.

| **Levenshtein distance** | **Procedure codes** | **Diagnostic codes** |
| --- | --- | --- |
| 0 | 287283 | 276952 |
| 1 | 2 | 1 |
| 2 | 44 | 6 |
| 3 |  | 23 |
| 4 | 10 | 516 |
| 5 | 3 | 9243 |
| 6 | 2 | 12 |
| 7 | 21 | 11 |
| 8 | 46 | 5 |
| 9 | 2 | 52 |
| 10 | 2 | 541 |
| 11 | 2 | 2 |
| 12 |  | 13 |
| 13 | 2 | 5 |
| 14 |  | 7 |
| 15 | 15 | 30 |
| 16 | 2 | 5 |
| 17 |  | 3 |
| 18 | 2 |  |
| 19 | 3 | 3 |
| 20 |  | 4 |
| 21 | 2 | 2 |
| 22 | 2 | 2 |
| 23 | 3 |  |
| 24 |  | 7 |
| 25 | 2 | 2 |
| 29 |  | 2 |
| 30 |  | 3 |
| 39 | 4 |  |
| 45 |  | 2 |
